# 30-Day Outcomes of Real-World Elective Carotid Stenosis Treatment Using a Dual-Layer Micromesh Stent (ROADSAVER Study)

**DOI:** 10.1007/s00270-025-04003-z

**Published:** 2025-03-19

**Authors:** Ralf Langhoff, Zsolt Vajda, Piotr Odrowąż-Pieniążek, Aleksandar Gjoreski, Roel Beelen, Koen Deloose, Balázs Nemes, Zoltán Ruzsa, Jean-Luc Banos, Sérgio Castro, Benjamin Faurie, Torsten Fuß, Michael Piorkowski, Istvan Király, Ivan Vulev, Arne Schwindt, Péter Csécsei, Alejandro Tomasello Weitz, Tomáš Jonszta, Paweł Latacz, Jorge Galván Fernández, Jürgen Verbist, Henrik Schröder, Christiane Pöckler-Schöniger, Karlis Kupcs, Pascual Lozano Vilardell, Rubén Rodríguez Carvajal, Kim Daenens, Matthias Tenholt, Peter Blaško, Olivier François, José Luis Diaz Valiño, Francisco Javier Martínez Gámez, Volker Sesselmann, Pál Bárzo, Wiebke Kurre, Mikel Terceño Izaga, Antonio Orgaz Pérez-Grueso, Karol Suppan, Jiří Lacman, José Angel Larrea Peña, Jordi Blasco, Reinoud Bokkers, Vladimir Cvetić, Viktor Till, Heliodoro Vallés González, Martin Andrassy, Daniel van den Heuvel, Jürgen Köhler, Stefan Müller-Hülsbeck, Sasko Kedev

**Affiliations:** 1 Department of Angiology, Brandenburg Medical School Theodor Fontane, Campus Clinic Brandenburg, Brandenburg an der Havel and Sankt Gertrauden – Hospital, Berlin, Germany; 2 Neurovascular Unit, Moritz Kaposi Teaching Hospital, Kaposvár, Hungary; 3 Department of Radiology, Fejér County Szent György University Teaching Hospital, Székesfehérvár, Hungary; 4 Department of Interventional Cardiology, Institute of Cardiology, Medical College, Jagiellonian University, Kraków, Poland; 5 Division on Endovascular Therapy, Department of Vascular Surgery, John Paul II Hospital, Kraków, Poland; 6 Department for Diagnostic and Interventional Radiology, Clinical Hospital “Acibadem Sistina”, Skopje, North Macedonia; 7 Department of Vascular and Thoracic Surgery, O.L.V. Aalst, Aalst, Belgium; 8 Department of Vascular Surgery, AZ-Sint Blasius, Dendermonde, Belgium; 9 Department of Interventional Radiology, Heart and Vascular Centre, Semmelweis University, Budapest, Hungary; 10 Bács-Kiskun County Hospital, Teaching Hospital of the Szent-Györgyi Albert Medical University, Kecskemét, Hungary; 11 Division of Invasive Cardiology, Department of Internal Medicine, University of Szeged, Szeged, Hungary; 12 Centre de Cardiologie et d’Exploration de la Côte Basque, Bayonne, France; 13 Interventional Neuroradiology Unit, Department of Imagiology, Centro Hospitalar Vila Nova de Gaia/Espinho, Vila Nova de Gaia, Portugal; 14 Infirmerie Protestante de Lyon, Caluire-et-Cuire, France; 15 Centre of Vasculare Medicine, Elblandklinikum, Radebeul, Radebeul, Germany; 16 Cardioangiologisches Centrum Bethanien at Agaplesion Bethanien Hospital, Frankfurt, Germany; 17 Központi Radiológiai Osztály, Markusovszky Egyetemi Oktatókórház Szombathely, Szombathely, Hungary; 18 Department of Interventional Radiology, CINRE. s.r.o., Bratislava, Slovakia; 19 Department of Vascular Surgery, St. Franziskus-Hospital, Münster, Germany; 20 Neuroendovascular Division, Department of Neurosurgery, University of Pécs, Pécs, Hungary; 21 Interventional Neuroradiology Section, Department of Radiology, Vall d’Hebron University Hospital, Barcelona, Spain; 22 Department of Radiology, University Hospital Ostrava, Ostrava, Czech Republic; 23 Faculty of Medicine, University of Ostrava, Ostrava, Czech Republic; 24 Department of Vascular Surgery and Angiology, Brothers of Mercy St. John of God Hospital, Kraków, Poland; 25 Department of Interventional Neuroradiology, Hospital Clínico Universitario de Valladolid, Valladolid, Spain; 26 Department of Vascular and Thoracic Surgery, Imelda Hospital Bonheiden, Bonheiden, Belgium; 27 Ihre-Radiologen.de, Center for Diagnostic Radiology and Minimally Invasive Therapy, The Jewish Hospital, Berlin, Germany; 28 Diagnostische Radiologie/Neuroradiologie, SRH Klinikum Karlsbad-Langensteinbach, Karlsbad, Germany; 29 Faculty of Medicine, Riga Stradiņš University, Riga, Latvia; 30 Department of Radiology, Riga Stradiņš University, Riga, Latvia; 31 Angiology and Vascular Surgery Department, Hospital Universitari Son Espases, Palma, Spain; 32 International Vascular and Endovascular Institute (IVEI), Angiology and Vascular Surgery Department, Hospital Quirónsalud Campo de Gibraltar, Palmones, Cádiz Spain; 33 Department of Vascular Surgery, University Hospitals Leuven, Leuven, Belgium; 34 Vein Center Pforzheim, Pforzheim, Germany; 35 Department of Interventional Cardiology, Kardiocentrum Nitra s.r.o., Nitra, Slovakia; 36 Department of Medical Imaging, AZ Groeninge, Kortrijk, Belgium; 37 Neuroradiology Department, Hospital Universitario A Coruña, A Coruña, Spain; 38 Servicio de Angiología y Cirugía Vascular, Complejo Hospitalario de Jaén, Hospital Universitario Médico-Quirúrgico, Jaén, Spain; 39 SRH Zentralklinikum Suhl, Klinik Für Innere Medizin I (Kardiologie, Angiologie Und Internistische Intensivmedizin), Suhl, Germany; 40 Neurosurgery Clinic, University of Szeged Hospital, Szeged, Hungary; 41 Department of Radiology and Neuroradiology, Klinikum Passau, Passau, Germany; 42 Stroke Unit. Department of Neurology, Hospital Dr Josep Trueta, Institut d’Investigació Biomèdica de Girona, Girona, Spain; 43 Servicio de Angiología y Cirugía Vascular, Hospital Universitario de Toledo, Toledo, Spain; 44 Clinic of Vascular and Internal Diseases, Dr. Jan Biziel University Hospital No. 2, Bydgoszcz, Poland; 45 Department of Radiology, Military University Hospital Prague, Prague, Czech Republic; 46 Interventional Neuroradiology Section, Department of Radiology, Donostia University Hospital, Donostia-San Sebastian, Spain; 47 Department of Interventional Neuroradiology, Hospital Clinic of Barcelona, Barcelona, Spain; 48 Department of Radiology, Medical Imaging Center, University Medical Center Groningen, University of Groningen, Groningen, The Netherlands; 49 Cardiovascular Radiology Department, Clinic for Vascular and Endovascular Surgery, University Clinical Centre of Serbia, Belgrade, Serbia; 50 Center of Radiology, Clinical Centre of Vojvodina, Novi Sad, Serbia; 51 Section of Vascular and Interventional Radiology, Department of Radiology, Hospital Universitario de Canarias, La Laguna, Tenerife Spain; 52 Fuerst-Stirum Hospital, Cardiology and Vascular Medicine, Bruchsal, Germany; 53 Department of Radiology, St Antonius Hospital, Nieuwegein, The Netherlands; 54 Clinic for Vascular and Endovascular Surgery, Pius-Hospital Oldenburg, Carl Von Ossietzky Universität, Oldenburg, Germany; 55 Department of Diagnostic and Interventional Radiology/Neuroradiology, Academic Teaching Hospital Christian-Albrechts-University Kiel – Faculty of Medicine, Deaconess Hospital Flensburg, DIAKO Hospital gGmbH, Knuthstraße 1, 24939 Flensburg, Germany; 56 Department of Cardiology, Faculty of Medicine, University Clinic of Cardiology, University of St. Cyril and Methodius, Skopje, North Macedonia

**Keywords:** Carotid artery stenting, Carotid artery disease, Carotid artery revascularization, Stroke prevention, Cerebrovascular embolic protection

## Abstract

**Purpose:**

Carotid artery stenting with single-layer stents carries a risk of periprocedural cerebral embolization compared to carotid endarterectomy. Dual-layer micromesh stents were designed for improved plaque coverage and sustained embolic protection. This analysis aimed to confirm the Roadsaver dual-layer micromesh stent safety in a real-world carotid artery stenting cohort.

**Materials and Methods:**

ROADSAVER was a prospective, single-arm, multicenter, observational study. Patients with carotid artery stenosis, eligible for elective stenting, were enrolled at 52 sites across 13 European countries. All procedures followed standard practice. The primary outcome was the 30-day major adverse event rate, defined as the cumulative incidence of any death or stroke. All deaths, strokes, and carotid artery revascularizations were independently adjudicated.

**Results:**

In total, 1965 patients were analysed (mean age 70.6 ± 8.8 years). Cerebral ischaemia symptoms were present in 49.4% of participants. Radial/ulnar access was used in 26.3% of cases and embolic protection in 63.8%. The 30-day major adverse event incidence was 2.2% (1.6% in asymptomatic and 2.8% in symptomatic patients), with any stroke at 1.9%, any death at 0.8%, and stroke-related death at 0.5%. Predictors of higher 30-day major adverse event risk, identified through multivariable modelling, included residual stenosis ≥ 30%, thromboembolic venous disease, previous myocardial infarction, age ≥ 75 years, family history of atherosclerosis, non-insulin-dependent diabetes mellitus, symptomatic carotid stenosis, and stent length.

**Conclusion:**

Dual-layer micromesh carotid artery stenting is safe, with a low 30-day major adverse event incidence in real-world asymptomatic and symptomatic patients, supporting the sustained embolic protection design concept.

**Level of Evidence:**

Level 2, observational study (with dramatic effect).

**Graphical Abstract:**

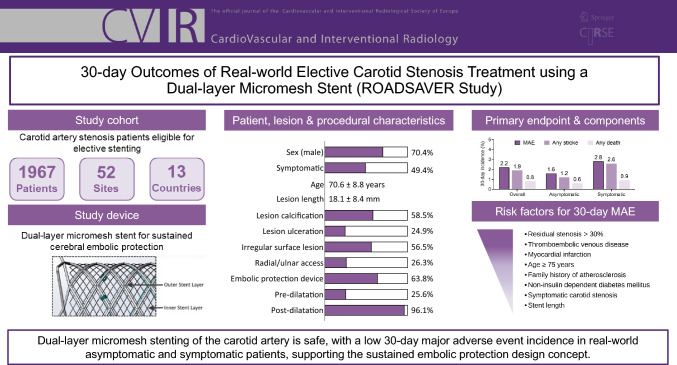

**Supplementary Information:**

The online version contains supplementary material available at 10.1007/s00270-025-04003-z.

## Introduction

Carotid artery stenosis accounts for up to 15% of all ischaemic strokes [1, 2]. Management options include lifestyle changes, medical therapy, surgical carotid endarterectomy (CEA), and endovascular carotid artery stenting (CAS). Randomized controlled trials (RCTs) using earlier-generation single-layer stents have shown slightly higher rates of 30-day periprocedural cerebrovascular events, mainly minor strokes, with CAS versus CEA, particularly in elderly, symptomatic patients [3]. New-generation dual-layer micromesh stent(s) (DLMS) were designed with an additional micromesh for better lesion coverage, limiting plaque prolapse through the stent struts and ensuring sustained cerebral embolic protection during and after the CAS procedure. Several studies have demonstrated the short- and long-term safety and efficacy of the DLMS [4–9]. This analysis expands the existing safety evidence to a real-world patient cohort, providing valuable insights into contemporary European clinical CAS practice.

## Materials and Methods

### Study Design and Population

The ROADSAVER study design has been previously described [10]. This prospective, single-arm, multicentre, observational study enrolled patients between January 2018 and February 2021 in 52 hospitals across 13 European countries. Eligibility criteria were minimal to evaluate the study device in a broad, real-world population. Patients with a non-occlusive and non-thrombotic, asymptomatic or symptomatic carotid artery stenosis, indicated for elective CAS, were enrolled. For more details on selection criteria, key definitions, study sites and investigators, see Supplemental Materials 1 and Supplemental Materials 2.

### Study Device

The Roadsaver DLMS (MicroVention Europe, a subsidiary of Terumo Corporation) is a braided, nickel-titanium (nitinol), self-expanding carotid stent with an internal micromesh (with 375–700 μm sized pores) and an outer layer with closed-cell design and flared ends. The stent (outer) diameter range includes sizes 5–10 mm and lengths 16–40 mm (22–47 mm with flares). The delivery system consists of a 5 Fr rapid-exchange catheter, which is 143 cm long and 0.014" guidewire compatible.

### Procedure

Baseline evaluations, diagnostic imaging and the CAS procedure followed routine hospital practice, including anticoagulation, other therapies, and operator-discretionary use of adjunctive devices and post-procedural antithrombotic therapy. Generally, dual antiplatelet therapy (DAPT: aspirin combined with a P2Y12 receptor inhibitor) was administered either prior to the CAS procedure or as an intraprocedural loading dose and continued for at least 1-month post-procedure. Oral anticoagulation, either alone or with single antiplatelet therapy or DAPT, was prescribed if indicated. Operators angiographically assessed lesions and quantified stenosis degree according to NASCET criteria [11] pre- and post-procedure. Follow-up occurred at 30 days (± 7 days) and 12 months (± 30 days); this analysis focuses on 30-day safety outcomes. For more details see [10].

### Outcome Measures and Definitions

The primary outcome measure was the 30-day major adverse event (MAE) rate, defined as the cumulative incidence of any death or stroke. Procedural outcomes included technical and procedural success rates, and device malfunctions. Clinical outcomes included 30-day incidences of death (any or stroke-related), stroke (any, major or minor), transient ischaemic attack (TIA), target lesion revascularization (TLR) and major vascular and bleeding complications (MVBC). Symptomatic patients were those who experienced amaurosis fugax ipsilateral to the carotid lesion, TIA, or non-disabling stroke within 180 days of the procedure within the hemisphere supplied by the target vessel. All deaths, strokes, and carotid artery revascularizations were adjudicated based on relevant source documents by an independent Clinical Events Committee composed of non-study physicians, see Supplemental Materials 1.

### Statistical Analyses

Sample size calculations for the study were based on achieving sufficient power to show non-inferiority in terms of the 30-day MAE rate compared with rates reported in prior CAS studies [10]. From these studies, a weighted mean 30-day MAE rate of 4.3% was calculated as the Objective Performance Criterion (OPC). Using a 1.3% non-inferiority delta, a 5.6% MAE rate was determined as the upper bound of the non-inferiority margin. A sample size of 2000 patients was calculated to provide > 80% power with a one-sided significance level of 0.05, assuming 7% attrition rate.

Continuous variables are represented using means and standard deviations (SD), while categorical variables are displayed as frequencies and percentages. The denominator for the primary endpoint incidence rate calculation included patients with either a MAE up to 30 days post-procedure or with follow-up data out to 30 days or beyond to confirm the absence of an event. For procedural and technical success rate calculation, all patients with an attempted study device implantation were included in the denominator. For secondary clinical endpoints, incidence rate calculations used a common denominator, including patients with any secondary outcome event or with sufficient data out to 30 days or beyond to confirm the absence of any event. Incidence rates are reported with 95% confidence intervals (CI), calculated using the Wilson score method.

A logistic regression analysis was carried out to investigate factors predicting MAE out to 30 days. A univariable model for each potentially explanatory factor was fitted, and those factors with a p-value < 0.1 were included in multivariable modelling using a stepwise selection process whereby explanatory variables were added to the model if the p-value was < 0.1 and removed if the p-value was > 0.1. All analyses were performed using SAS software, version 9.4 (SAS Institute Inc., Cary, NC, USA).

### Ethical Approval

The study was performed in accordance with the ethical standards of the institutional and/or national research committees and Helsinki declaration. The Institutional Review Board of each participating centre approved the study as per local regulations, and all patients provided written informed consent. The clinicaltrial.gov study identifier is NCT03504228.

## Results

### Baseline Patient and Lesion Characteristics

**Fig. 1:**
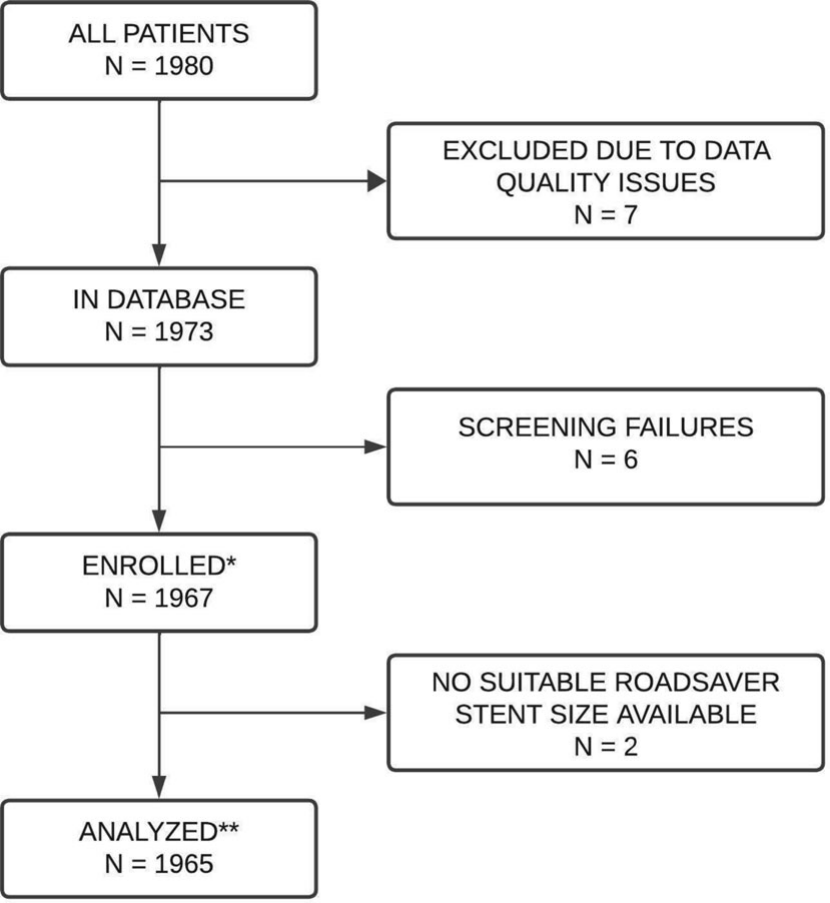
The patient disposition flow-chart. *Patients were enrolled if compliant with the eligibility criteria and after successful guidewire passage through the target lesion. **Analyses included 1965 enrolled patients who received the study device

A total of 1967 patients were enrolled. In two cases, however, other stents were used due to the lack of suitably sized study devices; these were not analysed, see Fig. 1. Among 1965 patients who received the study device, 49.4% were symptomatic. The mean age of the study population was 70.6 ± 8.8 years. Hypertension was noted in 87.4%, hyperlipidaemia in 76.0% and diabetes mellitus in 32.1% of the study participants. An aortic arch type I or II was present in 87.8% of the patients. The mean lesion length (as per operator assessment) was 18.1 ± 8.4 mm. For more details, see Table 1.

**Table 1:** Baseline patient and lesion characteristics

Patient characteristics	N = 1965
Demographic	
Age (years)	70.6 ± 8.8
Range (years)	30–95
≥ 75 years	35.9 (705)
Sex (male)	70.4 (1383)
Neurologic status	
Symptomatic	49.4 (971)
Asymptomatic	50.6 (994)
Medical history and risk factors	
Diabetes mellitus (DM)	32.1 (631)
Insulin-dependent DM	20.9 (132/631)
Noninsulin-dependent DM	79.1 (499/631)
Hyperlipidaemia	76.0 (1493)
Hypertension	87.4 (1718)
Obesity	23.2 (455)
Current smoker	25.2 (495/1962)
Previous smoker	41.7 (818/1962)
Cardiovascular disease	38.3 (752)
Peripheral vascular disease	26.8 (527)
Thromboembolic venous disease	2.2 (44)
Family history of atherosclerosis	14.5 (284)
Myocardial infarction	13.1 (257)
Cardiac arrhythmia	14.0 (276)
Valvular disease	7.4 (146)
Any intracranial pathology	5.3 (105)
Aortic arch anatomy	
Type I	54.4 (1069/1964)
Type II	33.4 (655/1964)
Type III	8.1 (160/1964)
Bovine	4.1 (80/1964)
Target lesion localization	
Right side	51.6 (1014)
Internal carotid artery/bifurcation	97.7 (1919)
Common carotid artery	2.3 (46)
Lesion characteristics*	
Lesion length (mm)	18.1 ± 8.4 (1964)
RVD-proximal (mm)	7.1 ± 1.2 (1879)
RVD-distal (mm)	5.0 ± 1.1 (1839)
Minimum lumen diameter (mm)	1.5 ± 0.9 (1870)
Calcification	58.5 (1149)
Ulceration	24.9 (489)
Concentricity	44.4 (840/1893)
Irregular surface	56.5 (1106/1957)
Severe (> 90°) target-vessel tortuosity	7.6 (150)

Values represent mean ± SD or % (n) as applicable. Summary statistics (means, SDs and percentages) are calculated based on the number of patients in the analysis set (N) with non-missing data, as indicated. Percentages for subjects without the condition or unknowns are not shown. *As per operator assessment. RVD: Reference Vessel Diameter; SD: Standard Deviation

### Procedural Characteristics

While femoral access predominated (70.3%), radial access (including 11 trans-ulnar cases) was used in 26.3% and a trans-cervical approach in 1.9% of the patients. Embolic protection was applied in 63.8% of the cases. Pre-dilatation was performed in 25.6% of the procedures. In total, 2002 study stents were implanted. Post-dilatation was performed in 96.1% of cases. Vascular closure devices were used in 63.9% of patients. For more details, see Table 2.

**Table 2:** Procedural characteristics

Procedural characteristics	N = 1965
**Access**	
Femoral	70.3 (1381)
Radial*	26.3 (516)
Cervical	1.9 (37)
Brachial	1.6 (31)
**Embolic protection**	
Embolic protection use	63.8 (1253)
Distal filter only	87.2 (1092/1253)
FilterWire EZ	53.0 (579/1092)
Spider FX	27.7 (303/1092)
Emboshield NAV6	19.0 (207/1092)
Other	0.3 (3/1092)
Proximal protection only	12.6 (158/1253)
Mo.Ma Ultra	76.6 (121/158)
Enroute NPS	22.8 (36/158)
FlowGate balloon guide catheter	0.6 (1/158)
Both distal and proximal protection	0.2 (3/1253)
Pre-dilatation	25.6 (504)
Post-dilatation	96.1 (1889)
**Stents**	
Single stent used	97.5 (1916)
> 1 stent used	2.5 (49)
> 1 Roadsaver DLMS used	75.5 (37/49)
Stent re-sheathed during implantation^**^	3.6 (72/2002)
Visible plaque protrusion via stent struts^***^	1.0 (19/1962)
VCD use	63.9 (1256)
Diameter stenosis (%)^***^ pre-procedure	80.2 ± 12.7
Diameter stenosis (%)^***^ post-procedure	7.0 ± 9.5

Values represent mean ± SD or % (n) as applicable. Summary statistics (means, SDs and percentages) are calculated based on the number of patients in the analysis set (N) with non-missing data, unless otherwise stated. Percentages for subjects without the condition or unknowns are not shown. *Including 11 cases of trans-ulnar artery access. **Percentage based on the total number of Roadsaver DLMS implanted (N = 2002). ***As per operator’s assessment, with the percentage diameter stenosis determined according to the angiographic NASCET criteria. DLMS: Dual-Layer Micromesh Stent; NASCET: North American Symptomatic Carotid Endarterectomy Trial; VCD: Vascular Closure Device; SD: Standard Deviation

### Procedural Outcomes

The technical success rate was 98.9% (95% CI: 98.3–99.3%). Technical failures occurred in 1.1% of subjects, including residual stenosis ≥ 30% in 1.0% (*n* = 20) and device malfunctions in 0.1% (*n* = 2) due to stent detachment issue and failure to advance the stent through the guiding catheter. The procedural success rate was 97.5% (95% CI: 96.7–98.1%).

### Clinical Outcomes at 30 Days

The incidence of the primary outcome measure of 30-day MAE was 2.2% (95% CI: 1.6–3.0%). The upper bound of the one-sided 95% CI of the 30-day MAE incidence was 2.8%, which is lower than the upper bound of the non-inferiority margin of 5.6%, confirming non-inferiority to the OPC. 30-day mortality was 0.8% (0.5% stroke-related). Incidence of any stroke was 1.9% (0.9% major / 1.0% minor). The 30-day rate of TIA was 0.9% and TLR incidence 0.8%. MVBCs were reported in 1.0% of patients. For more details, see Table 3.

**Table 3:** Clinical outcomes at 30 days

	N = 1965	
	Incidence rate	95% CI
Primary composite endpoint		
MAE	2.2 (43)	1.6–3.0
Secondary endpoints		
Any stroke	1.9 (37)	1.4–2.6
Major stroke	0.9 (18)	0.6–1.5
Minor stroke	1.0 (19)	0.6–1.5
Any death	0.8 (15)	0.5–1.3
Stroke-related death	0.5 (9)	0.2–0.9
TLR	0.8 (15)	0.5–1.3
TIA	0.9 (18)	0.6–1.5
MVBC	1.0 (20)	0.7–1.6

Values represent estimated incidence rates % (n) with 95% CI calculated using the Wilson score method for primary and secondary clinical outcomes. The percentages are based on the number of patients in the analysis set (N) with an event or with sufficient data out to 30 days or beyond to confirm its absence. A common denominator is used for all secondary endpoints. CI: Confidence Interval; MAE: Major Adverse Event (i.e. cumulative incidence of any death or stroke); MVBC: Major Vascular and Bleeding Complications; TIA: Transient Ischaemic Attack; TLR: Target Lesion Revascularization

The 30-day incidence of MAE was 1.6% in asymptomatic and 2.8% in symptomatic patients. The stroke-related death rate was low in both groups (0.2% and 0.7%, respectively). Any stroke incidence was 1.2% in asymptomatic and 2.6% in symptomatic patients, with major stroke rates of 0.6% and 1.2%, respectively. For more details, see Table 4.

**Table 4:** Clinical outcomes at 30 days in asymptomatic and symptomatic patients

	Asymptomatic N = 994	95% CI	Symptomatic N = 971	95% CI
Primary composite outcome				
MAE	1.6 (16)	1.0–2.6	2.8 (27)	1.9–4.0
Secondary endpoints				
Any death	0.6 (6)	0.3–1.3	0.9 (9)	0.5–1.8
Stroke-related death	0.2 (2)	0.1–0.7	0.7 (7)	0.4–1.5
Any stroke	1.2 (12)	0.7–2.1	2.6 (25)	1.8–3.8
Major stroke	0.6 (6)	0.3–1.3	1.2 (12)	0.7–2.2
Minor stroke	0.5 (5)	0.2–1.2	1.4 (14)	0.9–2.4

Values represent estimated incidence rates % (n) with 95% CI calculated using the Wilson score method for primary and selected secondary clinical outcomes (primary endpoint components) in asymptomatic and symptomatic patients. The percentages are based on the number of patients in subgroup analysis sets (N) with an event or with sufficient data out to 30 days or beyond to confirm its absence. A common denominator was used for all secondary endpoints. Among the symptomatic patients, one experienced a minor and a major stroke, while another had two major strokes. In one asymptomatic patient, it was impossible to adjudicate one stroke as minor or major. CI: Confidence Interval; MAE: Major Adverse Event (i.e. cumulative incidence of any death or stroke)

### Logistic Regression

**Fig. 2:**
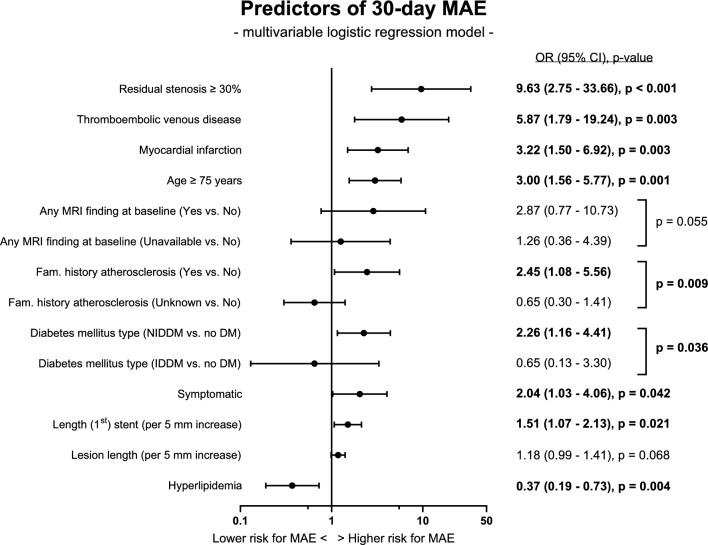
The forest plot summarizes the results of the multivariable logistic regression modelling, which identified several unique predictors of 30-day MAE. Odds ratios (with 95% CIs) and p-values of variables significant at 5% level are shown in bold. CI: Confidence Interval; IDDM: Insulin-Dependent Diabetes Mellitus; NIDDM: Non-Insulin-Dependent Diabetes Mellitus; OR: Odds Ratio

The univariable logistic regression analyses found potential risk factors for inclusion in the multivariable model of 30-day MAE (Supplemental Material 1; Table S1), which subsequently identified residual stenosis ≥ 30% (OR 9.63, 95% CI 2.75–33.66), thromboembolic venous disease (OR 5.87, 95% CI 1.79–19.24), myocardial infarction (MI) (OR 3.22, 95% CI 1.50–6.92), age ≥ 75 years (OR 3.00, 95% CI 1.56–5.77), family history of atherosclerosis (OR 2.45, 95% CI 1.08–5.56), non-insulin-dependent diabetes mellitus (NIDDM) (OR 2.26, 95% CI 1.16–4.41), symptomatic carotid stenosis (OR 2.04, 95% CI 1.03–4.06) and stent length (OR 1.51, 95% CI 1.07–2.13) as significant independent predictors of an increased risk of 30-day MAE and (medically managed) hyperlipidaemia associated with a lower 30-day MAE risk (OR 0.37, 95% CI 0.19–0.73). For a graphic representation of these results, see Fig. 2.

## Discussion

This real-world analysis demonstrates a low incidence of 30-day MAE in patients undergoing elective CAS with a DLMS, both in asymptomatic and symptomatic patients. These results were obtained from a heterogeneous cohort that reflects contemporary CAS practice, including complex vascular anatomies and lesions, and a liberal use of embolic protection devices.

Since carotid revascularization is performed to prevent stroke, short-term MAE are considered the most relevant outcome measure for assessing treatment safety. The 30-day MAE incidence of 2.2% in this study was non-inferior to the OPC of 5.6%, based on earlier single-layer stent studies. The results also compare favourably to guideline-recommended thresholds and outcomes of landmark RCTs. The reported 30-day MAE rates of 1.6% in asymptomatic and 2.8% in symptomatic patients are well below the European Society for Vascular Surgery (ESVS) 2023 guideline-recommended thresholds of 3% and 6%, respectively [2] Additionally, the results are below the more conservative German CAS guidelines, which mandate in-hospital MAE rates of 2% and 4% for both CEA and CAS in the two patient subsets, respectively [1]. Concerning RCTs, in the CREST study, the 30-day rate of any death or stroke in the CAS group was 4.4% overall, with rates of 2.5% for asymptomatic and 6.0% for symptomatic patients [12, 13]. In the ICSS RCT, which exclusively enrolled symptomatic patients, the 30-day MAE rate for the CAS group was 7.4%, higher than the 2.8% rate observed in symptomatic patients in the current study [14]. Finally, in the recent ACST2 RCT with only asymptomatic patients, the 30-day stroke or death rate for those undergoing CAS was 3.7% [15], higher than the 1.6% rate observed in the present study. While acknowledging the limited validity of inter-study comparisons, these exceptional real-world results suggest that the Roadsaver DLMS displays a better safety profile than the devices used in the studies that shaped current guidelines. However, sufficiently powered RCTs with hard endpoints may be needed to confirm its clinical benefits over single-layer stents.

According to a Cochrane systematic review and meta-analysis of available RCTs, CAS with single-layer stents is associated with a higher periprocedural (≤ 30 days) risk of stroke or death compared with CEA. This difference is primarily driven by higher rates of minor non-disabling strokes, especially in elderly (≥ 70 years old) symptomatic patients [3]. To overcome this complication, DLMS were designed to limit the risk of cerebral embolization during and after the procedure. The key innovative feature of the DLMS is the micromesh layer that provides improved plaque coverage and sustained embolic protection by reducing the risk of plaque prolapse through the stent struts. Several meta-analyses have evaluated the 30-day clinical performance of DLMS, concluding that they show promising safety profiles [4, 5, 9, 16]. For instance, a meta-analysis including 68,422 patients from 112 mostly single-arm studies comparing DLMS to first-generation single-layer carotid stents concluded that certain DLMS, including the one studied here, improve 30-day outcomes of CAS [9]. Moreover, a 2016 diffusion-weighted magnetic resonance imaging (DW-MRI) study evaluating the occurrence of silent ischaemic cerebral lesions 24 h after implanting the Roadsaver DLMS concluded that its use might result in lower microembolic event rates compared with conventional single-layer stents [17]. A 2019 study by the same group found no difference between this and another DLMS in terms of incidence and volume of silent cerebral infarctions detected by DW-MRI [18]. Moreover, the Roadsaver DLMS was associated with a lower embolization rate and embolic debris load relative to single-layer stents, especially with high-risk plaques [19]. Finally, an RCT using Transcranial Doppler ultrasound for real-time cerebral monitoring during the CAS procedure on high-risk plaques showed that the Roadsaver DLMS reduced microembolizations relative to a single-layer stent, especially when combined with the proximal embolic protection system [6].

In the present study, residual stenosis ≥ 30% and stent length were identified as procedural characteristics that independently predict a higher 30-day MAE risk. This underscores the importance of proper stent sizing and implantation for treatment safety. The high level of operator confidence in the DLMS technology to prevent periprocedural cerebral events is demonstrated by the fact that 96.1% of patients underwent post-dilatation. Among patient characteristics, the presence of thromboembolic venous disease, previous MI, age ≥ 75 years, family history of atherosclerosis, NIDDM and symptomaticity were identified as independent risk predictors. Some of these, like age and symptomaticity are well-established risk factors in CAS [3, 20], while others, such as previous MI and diabetes have been implicated as possible risk modifiers [21, 22]. Although the mechanism behind thromboembolic venous disease and familial history of atherosclerosis as risk factors is unclear, it can be speculated that genetic predisposition associated with these conditions may harbour factors which increase the risk of cerebrovascular complications. Interestingly, the presence of hyperlipidemia was associated with a lower 30-day MAE risk, potentially due to the prescribed use of high-dose statins, suggesting a possible protective role of lipid-lowering therapy. Although this hypothesis requires further testing, the protective role of statins in CAS has been reported previously [23]. Overall, these results corroborate some previous findings and identify new predictors of 30-day MAE following CAS with DLMS.

### Study Limitations

The observational nature of the study and the absence of a comparator arm limit the ability to draw definitive conclusions about the performance and safety of the investigated DLMS. However, the results should be viewed within the broader context of clinical data collected with DLMS, including imaging studies that demonstrated a reduction in embolization frequency and burden. The study aimed to document real-world practices without any per-protocol mandated requirements for patient work-up. Treatments were administered according to international guidelines and were potentially influenced by socio-economic factors, such as device availability and reimbursement levels, which could impact clinical outcomes. Lastly, no central core-laboratory was involved in image analysis; vessel and lesion characteristics were qualitatively classified, and stenosis degree was quantitatively measured at the operator’s discretion.

## Conclusions

In this real-world study of elective CAS, the use of Roadsaver DLMS resulted in a low incidence of 30-day MAE for both asymptomatic and symptomatic patients, supporting the DLMS design concept of sustained periprocedural embolic protection. Additionally, the analysis identified several independent predictors of 30-day MAE, which could aid in future patient selection and risk stratification.

## Supplementary Information

Below is the link to the electronic supplementary material.

Supplementary file1 (DOCX 72 KB)

Supplementary file2 (DOCX 69 KB)

## Abbreviations

CAS: Carotid artery stenting
CEA: Carotid endarterectomy
DAPT: Dual-antiplatelet therapy
DLMS: Dual-layer micromesh stent
EPD: Embolic protection device
MAE: Major adverse event
MI: Myocardial infarction
MVBC: Major vascular bleeding complication
NASCET: North American symptomatic carotid endarterectomy trial
OPC: Objective performance criterion
OR: Odds ratio
RCT: Randomized controlled trial
TIA: Transient ischaemic attack
TLR: Target lesion revascularization
