# Supplementary material for: 30-Day Outcomes of Real-World Elective Carotid Stenosis Treatment Using a Dual-Layer Micromesh Stent (ROADSAVER Study)
Source: Cardiovasc Intervent Radiol. 2025 Mar 19;48(4):427–37. doi: 10.1007/s00270-025-04003-z (PMC11958397; doi:10.1007/s00270-025-04003-z)
Supplement: Supplementary file 1 — Supplementary file1 (DOCX 72 KB) [file 270_2025_4003_MOESM1_ESM.docx]

**Supplemental Information**

30-day Outcomes of Real-world Elective Carotid Stenosis Treatment using a Dual-layer Micromesh Stent (ROADSAVER Study)

**Table of contents**

[Inclusion and exclusion criteria 1](#_Toc190694581)

[Key definitions 2](#_Toc190694582)

[Clinical Events Committee (CEC) Members with Affiliations 3](#_Toc190694583)

[Table S1: Results of univariable and multivariable logistic regression modelling of 30-day MAE 4](#_Toc190694584)

# Inclusion and exclusion criteria

**Inclusion criteria**

- The patient has a non-occlusive and non-thrombotic carotid artery stenosis
- Eligible to be treated with Roadsaver™ Carotid Stent as per the Instructions for Use
- At least 18 years of age
- Life expectancy of at least 12 months from the date of the index procedure
- Informed consent signed

**Exclusion criteria**

- Any condition that makes patient unsuitable for percutaneous transluminal angioplasty, including intolerance or allergy to any material used and accompanying therapy.

# Key definitions

**Stroke:** An acute neurologic event with focal symptoms and signs, lasting for 24 h or more.

**Minor Stroke:** A new neurological event that resolves completely within 7 days or increases the NIHSS by ≤4 points.

**Major Stroke** A new neurological event that persists for >24 h and results in a >4 point increase in the NIHSS score relative to baseline or any subsequent lower score.

**Target lesion revascularization (TLR):** Any revascularization procedure of the original treatment site, including angioplasty, stenting, endarterectomy, or thrombolysis, performed to open or increase the luminal diameter inside or within 5 mm of the previously treated lesion.

**Major vascular and bleeding complications** were defined as at least one of the following**:**

- Major hematoma, defined as one requiring transfusion, surgical evacuation, or delay in discharge
- Pseudo aneurysm or Arteriovenous fistula or Retroperitoneal bleeding
- Peripheral ischemia/nerve injury caused by the proximal access site
- Vascular surgical repair to correct a local vascular access site complication and bleeding

**Technical success** was defined as a successful access and deployment of the device with recanalization determined by less than 30% residual stenosis by angiography at the time of the index procedure.

**Procedural success** was defined as technical success without any device- or procedure-related death, stroke, or any other serious periprocedural adverse events (i.e., those occurring on the index procedure day).

**Device malfunction** was defined as the failure of a device after its introduction into the study subject (i.e., failure to perform in accordance with its intended purpose when used as per the IFU or the Clinical Investigation Plan (CIP).

# Clinical Events Committee (CEC) Members with Affiliations

| **CEC Member** | **Site** |
| --- | --- |
| Prof. Dr. Claude Hanet | CHU UCL Namur, Belgium |
| Prof. Dr. Klaus Mathias | Herdecke, Germany |
| Dr. Bas Schölzel | Amphia Hospital Breda, Netherlands |
| Dr. George Vlachojannis | Universitair Medisch Centrum Utrecht, Netherlands |

# Table S1: RESULTS OF UNIVARIABLE AND MULTIVARIABLE LOGISTIC REGRESSION MODELLING OF 30-DAY MAE

|  | **Univariable** | | | **Multivariable** | | |
| --- | --- | --- | --- | --- | --- | --- |
|  | **OR** | **95% CI** | **p-value** | **OR** | **95% CI** | **p-value** |
| **Patient characteristics** |  |  |  |  |  |  |
| Age ≥ 75 years  (vs. < 75 years) | 2.81 | 1.52–5.22 | **0.001** | 3.00 | 1.56–5.77 | **0.001** |
| Diabetes mellitus (DM) type  (IDDM vs. no DM) | 0.88 | 0.20–3.77 | **0.049** | 0.65 | 0.13–3.30 | **0.036** |
| DM type  (NIDDM vs. no DM) | 2.14 | 1.14–3.99 |  | 2.26 | 1.16–4.41 |  |
| Hyperlipidaemia  (Yes vs. No) | 0.47 | 0.25–0.88 | **0.018** | 0.37 | 0.19–0.73 | **0.004** |
| Myocardial infarction  (Yes vs. No) | 2.64 | 1.34–5.22 | **0.005** | 3.22 | 1.50–6.92 | **0.003** |
| Thromboembolic venous disease  (Yes vs. No) | 4.80 | 1.64–14.07 | **0.004** | 5.87 | 1.79–19.24 | **0.003** |
| Family history of atherosclerosis  (Yes vs. No) | 2.57 | 1.23–5.36 | **0.017** | 2.45 | 1.08­–5.56 | **0.009** |
| Family history of atherosclerosis  (Unknown vs. No) | 0.95 | 0.46–1.96 |  | 0.65 | 0.30–1.41 |  |
| Any MRI finding at baseline  (Yes vs. No) | 2.83 | 0.81–9.98 | **0.067** | 2.87 | 0.77–10.73 | 0.055 |
| Any MRI finding at baseline  (MRI Unavailable vs. No) | 1.39 | 0.42–4.63 |  | 1.26 | 0.36–4.39 |  |
| Symptomatic  (Yes vs. No) | 1.75 | 0.94–3.26 | **0.080** | 2.04 | 1.03–4.06 | **0.042** |
| Age  (per year increase) | 1.05 | 1.01–1.09 | **0.014** |  |  |  |
| DM (Yes vs. No) | 1.87 | 1.02–3.43 | **0.044** |  |  |  |
| Cardiac arrhythmia  (Yes vs. No) | 2.15 | 1.07–4.33 | **0.031** |  |  |  |
| Any intracranial pathology  (Yes vs No) | 2.41 | 0.93–6.25 | **0.071** |  |  |  |
| Hypertension  (Yes vs No) | 0.89 | 0.37–2.12 | 0.785 |  |  |  |
| Sex  (Female vs. Male) | 1.28 | 0.68 –2.42 | 0.442 |  |  |  |
| Peripheral vascular disease  (Yes vs. No) | 0.83 | 0.41–1.70 | 0.615 |  |  |  |
| Cardiovascular disease  (Yes vs. No) | 0.96 | 0.51–1.79 | 0.895 |  |  |  |
| Extracranial supra-aortic disease  (Yes vs. No) | 1.12 | 0.61–2.09 | 0.712 |  |  |  |
| Valvular heart disease  (Yes vs. No) | 0.29 | 0.04–2.14 | 0.226 |  |  |  |
| Obesity  (Yes vs. No) | 1.14 | 0.57–2.27 | 0.719 |  |  |  |
| Smoking  (Current vs. Never) | 0.69 | 0.29–1.64 | 0.588 |  |  |  |
| Smoking (Former vs. Never) | 1.05 | 0.54–2.08 |  |  |  |  |
| **Lesion and procedure characteristics** |  |  |  |  |  |  |
| Lesion length  (per 5 mm increase) | 1.17 | 1.00–1.36 | **0.052** | 1.18 | 0.99–1.41 | 0.068 |
| Stent length (1^st^ stent) (per 5 mm increase) | 1.37 | 1.00–1.88 | **0.048** | 1.51 | 1.07–2.13 | **0.021** |
| Residual stenosis ≥ 30% (vs. < 30%) | 12.15 | 3.88–38.01 | **<0.001** | 9.63 | 2.75–33.66 | **<0.001** |
| Diameter stenosis post-procedure (per 5% increase) | 1.17 | 1.02–1.35 | **0.024** |  |  |  |
| Pre-dilatation balloon size (≤ 3 mm vs. > 3 mm) | 0.36 | 0.12–1.07 | **0.069** |  |  |  |
| Pre-dilatation balloon size (No-predilatation vs. > 3 mm) | 0.40 | 0.18–0.90 |  |  |  |  |
| Post-dilatation  (Yes vs. No) | 0.38 | 0.13–1.10 | **0.074** |  |  |  |
| Post-dilatation balloon pressure (≤ 11 atm vs. > 11 atm) | 1.81 | 0.82–3.99 | **0.067** |  |  |  |
| Post-dilatation balloon pressure (No post-dilatation vs. > 11 atm) | 4.20 | 1.23–14.30 |  |  |  |  |
| Arterial access  (Femoral vs. Radial [incl. ulnar]) | 1.24 | 0.61–2.53 | 0.559 |  |  |  |
| Embolic protection device use  (Yes vs. No) | 1.68 | 0.84–3.36 | 0.141 |  |  |  |
| Diameter stenosis pre-procedure  (per 5% increase) | 1.03 | 0.91–1.16 | 0.689 |  |  |  |
| Pre-dilatation  (Yes vs. No) | 1.42 | 0.74–2.70 | 0.292 |  |  |  |
| Pre-dilatation balloon pressure  (≤ 9 atm vs. > 9 atm) | 0.55 | 0.18–1.70 | 0.363 |  |  |  |
| Pre-dilatation balloon pressure  (No pre-dilatation vs. > 9 atm) | 0.57 | 0.26–1.26 |  |  |  |  |
| Total stent length  (per 1 mm increase) | 1.03 | 0.99–1.08 | 0.116 |  |  |  |
| Post-dilatation balloon size  (≤4.5 mm vs. 5.5 mm) | 1.20 | 0.46–3.14 | 0.257 |  |  |  |
| Post-dilatation balloon size  (≤ 5.5 mm vs. > 5.5 mm | 0.81 | 0.39–1.69 |  |  |  |  |
| Post-dilatation balloon size  (No post-dilatation vs. > 5.5 mm) | 2.38 | 0.74–7.69 |  |  |  |  |
| Stent re-sheathed (Yes vs. No) | 2.00 | 0.60–6.63 | 0.256 |  |  |  |
| Visible plaque protrusion  (Yes vs. No) | 2.41 | 0.31–18.52 | 0.459 |  |  |  |
| Visible plaque protrusion  (Unknown vs. No) | 0.40 | 0.05–2.95 |  |  |  |  |

Values represent odds ratios with 95% Wald confidence intervals for factors potentially predictive of MAE out to 30 days. Note that where counts were low (<100) in the unknown categories, these have been collapsed into the "No" category for improved model fit. Any variable in the univariable analysis (left) with a p-value <0.1 (marked in bold) was fitted into a multivariable model. Variables retained in the multivariable model (right) identify unique predictors of 30-day MAE; p-values marked in bold highlight variables significant at 5% level. CI: Confidence Interval; DM: Diabetes Mellitus; IDDM: Insulin-Dependent Diabetes Mellitus; NIDDM: Non-Insulin-Dependent Diabetes Mellitus; OR: Odds Ratio.
