# Supplementary material for: 30-Day Outcomes of Real-World Elective Carotid Stenosis Treatment Using a Dual-Layer Micromesh Stent (ROADSAVER Study)
Source: Cardiovasc Intervent Radiol. 2025 Mar 19;48(4):427–37. doi: 10.1007/s00270-025-04003-z (PMC11958397; doi:10.1007/s00270-025-04003-z)
Supplement: Supplementary file 2 — Supplementary file2 (DOCX 69 KB) [file 270_2025_4003_MOESM2_ESM.docx]

30-day Outcomes of Real-world Elective Carotid Stenosis Treatment using a Dual-layer Micromesh Stent (ROADSAVER Study)

**Participating sites and (co)investigators**

| **Country** | **Site** | **Investigator(s)** |
| --- | --- | --- |
| Belgium | Department of Vascular and Thoracic Surgery Imelda Hospital Bonheiden Bonheiden | Jürgen Verbist (PI) Wouter Van Den Eynde |
| Belgium | Department of Medical Imaging AZ Groeninge Kortrijk | Olivier François (PI) Tommy Andersson |
| Belgium | Department of Vascular Surgery University Hospitals Leuven Leuven | Kim Daenens (PI) Sabrina Houthoofd |
| Belgium | Department of Vascular and Thoracic Surgery O.L.V. Aalst Aalst | Roel Beelen (PI) Maene Lieven Moerman Leslie Isabel Bouckenooghe |
| Belgium | Department of Vascular Surgery AZ-Sint Blasius Dendermonde | Koen Deloose (PI) Joren Callaert |
| Czech Republic | Department of Radiology University Hospital Ostrava Ostrava  Faculty of Medicine University of Ostrava Ostrava | Tomáš Jonszta (PI) Václav Procházka |
| Czech Republic | Department of Radiology Military University Hospital Prague Prague | Jiří Lacman (PI) |
| France | Centre de Cardiologie et d'Exploration de la Côte Basque Bayonne | Jean-Luc Banos (PI) Valerico Sanchez |
| France | Institut Cardiovasculaire Grenoble | Benjamin Faurie (PI) Serge Lanternier Carmine Sessa Dominique Chaix |
| Germany | Centre of Vasculare Medicine Elblandklinikum Radebeul | Torsten Fuß |
| Germany | SRH Zentralklinikum Suhl Klinik für Innere Medizin I (Kardiologie Angiologie und internistische Intensivmedizin) Suhl | Volker Sesselmann (PI) Torsten Vogel Tina Zahlaus Albert Woratcheck |
| Germany | Fuerst-Stirum Hospital Cardiology and Vascular Medicine Bruchsal | Martin Andrassy (PI) Katrin Gegenheimer |
| Germany | Clinic for Vascular and Endovascular Surgery,  Pius-Hospital Oldenburg,  Carl von Ossietzky Universität,  Oldenburg | Jürgen Köhler (PI) Christophe-Maria Ratusinski (Former PI) Andreas Cöster |
| Germany | Department of Diagnostic and Interventional Radiology and Neuroradiology DIAKO Hospital gGmbH Flensburg | Stefan Müller-Hülsbeck (PI) Michael Preiss Leonardo Marques Silke Hopf-Jensen |
| Germany | Department of Radiology and Neuroradiology Klinikum Passau Passau | Wiebke Kurre (PI) |
| Germany | Cardioangiologisches Centrum Bethanien at Agaplesion Bethanien Hospital Frankfurt | Michael Piorkowski (PI) Vladislav Ganchev |
| Germany | Ihre-Radiologen.de Center for Diagnostic Radiology & Minimally Invasive Therapy The Jewish Hospital Berlin | Henrik Schröder (PI) Ferdinand Rücker Alexandre Lucas |
| Germany | Abteilung Gefäß- und Endovascular Chirurgie Theresienkrankenhaus und Sankt Hedwig-Klinik Abteilung für Gefäßchirurgie Mannheim | Matthias Tenholt (PI) Maher Fattoum Hamad Algedaiby Domenico Marco Stillitano |
| Germany | Diagnostische Radiologie/Neuroradiologie SRH Klinikum Karlsbad-Langensteinbach Karlsbad | Christiane Pöckler-Schöniger (PI) Fritz Bergen |
| Germany | Department of Vascular Surgery St. Franziskus-Hospital Münster | Arne Schwindt (PI) Giovanni Torsello Michel Bosiers Thomas Schönefeld |
| Germany | Department of Angiology Brandenburg Medical School Theodor Fontane Campus Clinic Brandenburg Brandenburg an der Havel & Sankt Gertrauden – Hospital Berlin | Ralf Langhoff (PI) Andrea Behne Mehmet Boral David Hardung |
| Hungary | Központi Radiológiai Osztály Markusovszky Egyetemi Oktatókórház Szombathely | Istvan Király (PI) Csaba Nagy |
| Hungary | Neurosurgery Clinic University of Szeged Hospital Szeged | Pál Barzó (PI) Tamas Nemeth  Markos-Gergely Gellerd |
| Hungary | Neurovascular Unit Moritz Kaposi Teaching Hospital Kaposvár  Department of Radiology Fejér County Szent György University Teaching Hospital, Székesfehérvár | Zsolt Vajda (PI) Monika Szöts Nagy Csaba Balazs Zsuzsa Danyi |
| Hungary | Department of Interventional Radiology Heart and Vascular Centre Semmelweis University Budapest | Balázs Nemes (PI) |
| Hungary | Bács-Kiskun County Hospital Teaching Hospital of the Szent-Györgyi Albert Medical University Kecskemét  Department of Internal Medicine Division of Invasive Cardiology University of Szeged Szeged | Zoltán Ruzsa (PI) Csavajda Ádám János |
| Hungary | Department of Neurosurgery Neuroendovascular Division University of Pécs Pécs | Péter Csécsei (PI) Alex Szolics (Former PI) |
| Latvia | Faculty of Medicine Riga Stradiņš University Riga  Department of Radiology Riga Stradiņš University Riga | Karlis Kupcs (PI) Helmut Kidikas |
| Netherlands | Department of Radiology Medical Imaging Center University Medical Center Groningen University of Groningen Groningen | Reinoud Bokkers (PI) Maarten Uyttenboogaart |
| Netherlands | Department of Radiology St Antonius Hospital Nieuwegein | Daniel van den Heuvel (PI) Jan Albert Vos Marc van Leersum |
| North Macedonia | Department for Diagnostic and Interventional Radiology Clinical Hospital ”Acibadem Sistina” Skopje | Aleksandar Gjoreski (PI) Filip Risteski |
| North Macedonia | Department of Cardiology Faculty of Medicine University Clinic of Cardiology University of St. Cyril & Methodius Skopje | Sasko Kedev (PI) Ivan Vasilev Danica Petkoska |
| Poland | Department of Interventional Cardiology Institute of Cardiology Jagiellonian University Medical College Krakow  Department of Vascular Surgery Division on Endovascular Therapy John Paul II Hospital Krakow | Piotr Odrowąz-Pieniążek (PI) Piotr Paluszek  Roman Machnik Marcin Misztal |
| Poland | Vascular Surgery Clinical Department University Hospital  Krakow | Paweł Latacz (PI) |
| Poland | Clinic of Vascular and Internal Diseases Dr. Jan Biziel University Hospital No. 2 Bydgoszcz | Karol Suppan (PI) |
| Portugal | Department of Imagiology Interventional Neuroradiology Unit Centro Hospitalar Vila Nova de Gaia/Espinho Vila Nova de Gaia | Sérgio Castro (PI) Manuel Ribeiro Miguel Veloso Pedro Barros Marta Rodrigues Sofia Figueiredo |
| Serbia | Cardiovascular Radiology Department  Clinic for Vascular and Endovascular Surgery University Clinical Centre of Serbia Belgrade | Momčilo Čolić (PI) Vladimir Cvetić  Borivoje Lukić |
| Serbia | Center of Radiology Clinical Centre of Vojvodina Novi Sad | Viktor Till (PI) Dragan Andjelic Vedran Zigic |
| Slovakia | Department of Interventional Cardiology Kardiocentrum Nitra s.r.o. Nitra | Peter Blaško (PI) Peter Kurray |
| Slovakia | Department of Interventional Radiology CINRE s.r.o. Bratislava | Ivan Vulev (PI)  Tibor Balázs |
| Spain | Interventional Neuroradiology Section Department of Radiology Vall d’Hebron University Hospital Barcelona | Alejandro Tomasello Weitz (PI) Marc Ribó David Hernandez |
| Spain | Department of Interventional Neuroradiology Hospital Clínico Universitario de Valladolid Valladolid | Jorge Galván Fernández (PI) Miguel Arturo Schuller Arteaga Mario Martinez Galdámez Mercedes de Lera Alfonso |
| Spain | Angiology and Vascular Surgery Department Hospital Universitari Son Espases Palma | Pascual Lozano Vilardell (PI) Krystell Daniela Escoto |
| Spain | International Vascular and Endovascular Institute (IVEI) Angiology and Vascular Surgery Department Hospital Quirónsalud Campo de Gibraltar Palmones (Cádiz) | Rubén Rodríguez Carvajal (PI) Fernando Gallardo |
| Spain | Neuroradiology Department Hospital Universitario A Coruña A Coruña | José Luis Diaz Valiño (PI) Enrique Buceta (Former PI) |
| Spain | Servicio de Angiología y Cirugía Vascular Complejo Hospitalario de Jaén Hospital Universitario Médico-Quirúrgico Jaén | Francisco Javier Martínez Gámez (PI) José Enrique Mata Campos Elena Herrero Martinez |
| Spain | Stroke Unit. Department of Neurology Hospital Dr Josep Trueta Institut d'Investigació Biomèdica de Girona Girona | Mikel Terceño Izaga (PI) Saima Bashir Laura Paul Joaquín Serena Yolanda Silva |
| Spain | Servicio de Angiología y Cirugía Vascular Hospital Universitario de Toledo Toledo | Antonio Orgaz Pérez-Grueso (PI) Maria Pilar Lamarca Mendoza |
| Spain | Interventional Neuroradiology Section Department of Radiology Donostia University Hospital Donostia-San Sebastian | José Angel Larrea Peña (PI) Pedro Navia Alvarez Javier Masso Romero Alexandre Lüttich Uroz |
| Spain | Department of Interventional Neuroradiology Hospital Clinic of Barcelona Barcelona | Jordi Blasco (PI) Juan Macho Fernandez (Former PI) Antonio Lopez Javier Moreno Luis San Román |
| Spain | Department of Radiology Section of Vascular and Interventional Radiology Hospital Universitario de Canarias La Laguna Tenerife | Heliodoro Vallés González (PI) Jorge Senkichi Uchiyamada |
